# Experiences of nursing students affected by covid-19 during face to face education: qualitative research

**DOI:** 10.1590/1980-220X-REEUSP-2024-0146en

**Published:** 2025-01-31

**Authors:** Hilal Dünmez, Gülnur Akkaya

**Affiliations:** 1 Ayvalık State Hospital, Ayvalık, Balıkesir, Turkey.; 2 Çanakkale Onsekiz Mart University, Faculty of Health Sciences, Department of Nursing, Çanakkale, Turkey.

**Keywords:** COVID-19, Students, Nursing, Qualitative Research, COVID-19, Estudantes de Enfermagem, Pesquisa qualitativa, COVID-19, Estudiantes de Enfermería, Investigación Cualitativa

## Abstract

**Objective:**

This study was conducted to determine the experiences of nursing students who received face-to-face education during the COVID-19 pandemic and contracted COVID-19.

**Method:**

A phenomenological qualitative study was used to understand the students’ experiences, and the study was completed with 11 students who volunteered. Semi-structured online interviews were conducted using an interview guide, and the interviews were recorded. Thematic analysis was employed to generate themes. MAXQDA 2022 software was used for the analysis of the research.

**Results:**

As a result of the research, 3 themes; themes are as follows: “COVID-19 disease process” with the sub-themes “emotions” and “gains”; “Educational process” with the sub-themes “technological problems”, financial constraints”, and “legal issues”; “nursing profession and values” with the sub-themes “importance of nursing”, “intense and challenging working environment”.

**Conclusion:**

According to the experiences of students who contracted Covid-19 disease during face-to-face education during the Covid-19 epidemic, it was concluded that nursing students should be provided with the necessary technological support, the legal regulations of universities should be revised, and psychosocial support should be provided to students.

## RESUMO

**Objetivo:**

Este estudo foi realizado para determinar as experiências de estudantes de enfermagem que receberam ensino presencial durante a pandemia de COVID-19 e contraíram COVID-19.

**Métodos:**

Foi realizado um estudo qualitativo fenomenológico para compreender as experiências dos estudantes, e o estudo foi concluído com 11 estudantes que se voluntariaram. Foram realizadas entrevistas semi-estruturadas em linha, utilizando um guião de entrevista, e as entrevistas foram gravadas. A análise temática foi utilizada para gerar temas. O software MAXQDA 2022 foi utilizado para a análise da pesquisa.

**Resultados:**

Como resultado da pesquisa, existem 3 temas: “Processo da doença COVID-19” com os subtemas “emoções” e “ganhos”; “Processo educacional” com os subtemas “problemas tecnológicos”, “restrições financeiras” e “questões legais”; “Profissão e valores de enfermagem” com os subtemas “importância da enfermagem” e “ambiente de trabalho intenso e desafiador”

**Conclusão:**

De acordo com as experiências dos estudantes que contraíram a doença Covid-19 no ensino presencial durante a epidemia de Covid-19, concluiu-se que os estudantes de enfermagem devem receber o apoio tecnológico necessário, os regulamentos legais das universidades devem ser revistos e deve ser prestado apoio psicossocial aos estudantes.

## RESUMEN

**Objetivo:**

Este estudio se realizó para determinar las experiencias de los estudiantes de enfermería que recibieron formación presencial durante la pandemia de la COVID-19 y contrajeron la enfermedad.

**Método:**

Se utilizó un estudio fenomenológico cualitativo para comprender las experiencias de los estudiantes, y el estudio se completó con 11 estudiantes que se ofrecieron como voluntarios. Las entrevistas en línea semiestructuradas se realizaron utilizando una guía de entrevistas y se grabaron. Se empleó el análisis temático para generar temas. Para el análisis de la investigación se utilizó el *software* MAXQDA 2022.

**Resultados:**

Como resultado de la investigación, 3 temas; los temas son los siguientes: «proceso de la enfermedad COVID-19» con los subtemas «emociones» y «ganancias»; «proceso educativo» con los subtemas «problemas tecnológicos», restricciones financieras y «cuestiones legales»; «profesión y valores de enfermería» con los subtemas «importancia de la enfermería», «ambiente de trabajo intenso y desafiante ».

**Conclusión:**

De acuerdo con las experiencias de los estudiantes que contrajeron la enfermedad Covid-19 durante la educación presencial durante la epidemia de Covid-19, se concluyó que se debe proporcionar a los estudiantes de enfermería el apoyo tecnológico necesario, se debe revisar la normativa legal de las universidades y se debe brindar apoyo psicosocial a los estudiantes.

## INTRODUCTION

Training qualified nurses, one of the most important problems during the pandemic process experienced in the world and in our country, is an issue that needs to be addressed seriously. The first Covid-19 case in Turkey was reported on March 11, 2020, and it rapidly spread^(1)^. Due to the rapid spread of the outbreak in Turkey, the Council of Higher Education decided on April 1, 2020, to switch to distance education for the 2019–2020 spring semester, and many departments of the universities transitioned to online education^(2)^. It has been reported by the Council of Higher Education that students who are in the graduation phase of nursing programs can complete their practical training in health units as well as through distance education. In the meeting of the Council of Deans of Nursing Faculties (HEMDEK), this decision of the Council of Higher Education was evaluated and it was suggested that the spring term education of students who are in the graduation phase be carried out through distance education.In line with this suggestion, the practices of senior students in nursing faculties/departments were carried out using methods such as simulation training, skill videos, virtual classroom, project, and case analysis.It was planned that the practical courses of 1st, 2nd and 3rd year nursing students (such as Fundamentals of Nursing, Surgical Diseases Nursing, Child Health and Diseases Nursing, Women’s Health and Diseases Nursing) would be extended according to the decision of the Council of Higher Education and the suggestion of HEMDEK or completed in a clinical/field environment at the beginning or during the next academic term. However, the Council of Higher Education has decided that the final exams will not be held face-to-face, but will be held through digital means or alternative methods. For this reason, the applications of these courses were also carried out through distance education^(3)^. Due to the clinical and theoretical nature of nursing curriculum, it was one of the most affected groups during the Covid-19 pandemic. Both teaching staff and students faced numerous challenges as classes and practices moved online. While students were subjected to intense stress due to the pandemic, they also struggled with the educational process^(4)^. Higher education institutions are responsible for defining the guidelines for nursing students and assessing the conditions^(5)^. Despite efforts to respond to needs through rapid adjustments, the suspension of face-to-face education meant that nursing students could not receive clinical training. This situation significantly impacted nursing education as the planned knowledge and skills for students could not be realized^(6,7,8,9,10,11,12,13)^.

The differentiation of teaching methods and transition to distance education during the Covid-19 pandemic posed significant challenges for students. Assessments conducted through different methods led to confusion, with some students’ grades increasing while others decreased^(10)^. The importance of this study is to determine the experiences of nursing students who received face-to-face education during the Covid-19 pandemic and caught Covid-19. No such study has been found in the literature. To the best of our knowledge, this is the first such study, and its original value is highly significant.

## METHODS

### Purpose

This study employs a phenomenological approach to explore the experiences of nursing students who contracted Covid-19 during face-to-face education.

Research Type: The research is qualitative and a phenomenological study. The chosen approach, in accordance with Lambert and Lambert^(14)^, was employed to offer insights into the perspectives of the participants. The reporting process of the study adhered to the COREQ (CONsolidated criteria for Reporting Qualitative research) guidelines, as outlined by Tong et al.^(15)^.

### Research Team and Reflexivity

One of the researchers was a faculty member in the nursing department at a university, and the other was both a graduate student and a nurse at a hospital. The second author works at the school where the research was conducted.Both researchers had taken a qualitative research course and were experienced in this field. The second author has an article on qualitative research published in the web of science.The authors worked during the Covid-19 pandemic and had similar experiences to the participants. No comments were added to the responses given by the participants in the research; the experiences of the participants were conveyed entirely.

### Settings and Participants

To enhance transferability, purposive sampling method was used in the study. In purposive sampling, participants are selected to have characteristics suitable for the research topic. In this study, the nursing students who had contracted Covid-19 when face-to-face education resumed at universities were targeted. This way, it was ensured that the participants’ views represented the research topic. In this study, the criteria proposed by Guba and Lincoln were used for trustworthiness^(16,17)^. In this context, for ensuring credibility, the number of participants was determined as 11. The population of the study consists of 32 nursing students who were enrolled in the nursing department of a university. The sample includes 11 nursing students who volunteered to participate in the research.

### Data Collection and Trustworthiness

This number (11 nursing students) is considered sufficient for an in-depth examination of the research topic. Each interview lasted approximately 35–40 minutes, and efforts were made to ensure that the interviews took place in a conversational atmosphere. This allowed for a thorough and comprehensive examination of the participants’ views. After obtaining the necessary permissions for the research, those students who had contracted Covid-19 during face-to-face education were informed about the purpose and content of the study. Appointments were made with the students for interviews, and the researcher conducted the interviews via smartphone at the scheduled time, recording the audio. Names were kept anonymous. Interviews with the participants in the research group continued until data saturation was reached and no new data were obtained.Notes were taken during the interview, and participants were asked questions again to verify. The interviews were written down on paper, and when repetitive statements began to occur, for example; my anxiety increased, I was constantly washing my hands, isolation was difficult, and no new statements were made, the interviews reached saturation. The data collection process continued until data saturation was achieved, meaning that no new information was being identified^(18)^. The data were stored on Google Drive™ accessible to both authors for security purposes.

Questions for the semi-structured interview:

How did you feel when you found out that you had Covid-19, and did you experience any gains during this process?

What were your educational experiences during the Covid-19 pandemic?

How did your perspective on the nursing profession change when you had Covid-19, and what did you think about pursuing a career in nursing in the future?

### Analysis and Evaluation of Data

Individual interviews were transcribed into computerized transcripts by the researcher. In data analysis, the following steps were followed: decoding the recorded interviews, repeated reading, creating units of meaning, extracting themes and subthemes, and revealing the phenomenon of interest from the participants’ perspectives^(17)^. The data analysis process followed a systematic inductive approach to clarify the sub-problems of the research. The interview questions were used as umbrella categories, and the answers to these questions formed subcategories. Since these subcategories were derived from the data, they represented all thoughts and comments expressed during the interviews. Through the coding method, the data were segmented into subcategories, sometimes at the level of sentences and sometimes at the level of paragraphs, until no new category emerged. These initial categories were then reclassified, and the irrelevant ones were removed to move to a higher level of categorization. The codes generated inductively were rearranged and placed under categories. This allowed for the creation of the categories directly from the data rather than coding directly into the categories. The entire dataset was transferred to the MAXQDA 20 program, and the coding and categorization of the interview transcripts were conducted more systematically compared to manual analysis. As the participants’ expressions could be easily seen, variations could be made during the reporting stage. Describing, explaining, and presenting the data in a way understandable to the reader was facilitated. In the reporting phase, categories were explained, descriptions were made, and findings were interpreted. The results obtained were also compared with those of similar studies in the literature. During the reporting phase, the records were read by the researchers multiple times, and decisions were made about which quotations would be appropriate to include in the report.

### The Ethical Aspect of the Research

Permission was obtained from the school administration before starting the data collection process. Written permission was obtained from the Scientific Research Ethics Board of a university (2022-YÖNP-0126). In addition, consent was obtained from the students who agreed to participate in the research.

## RESULTS

**Figure 1:**
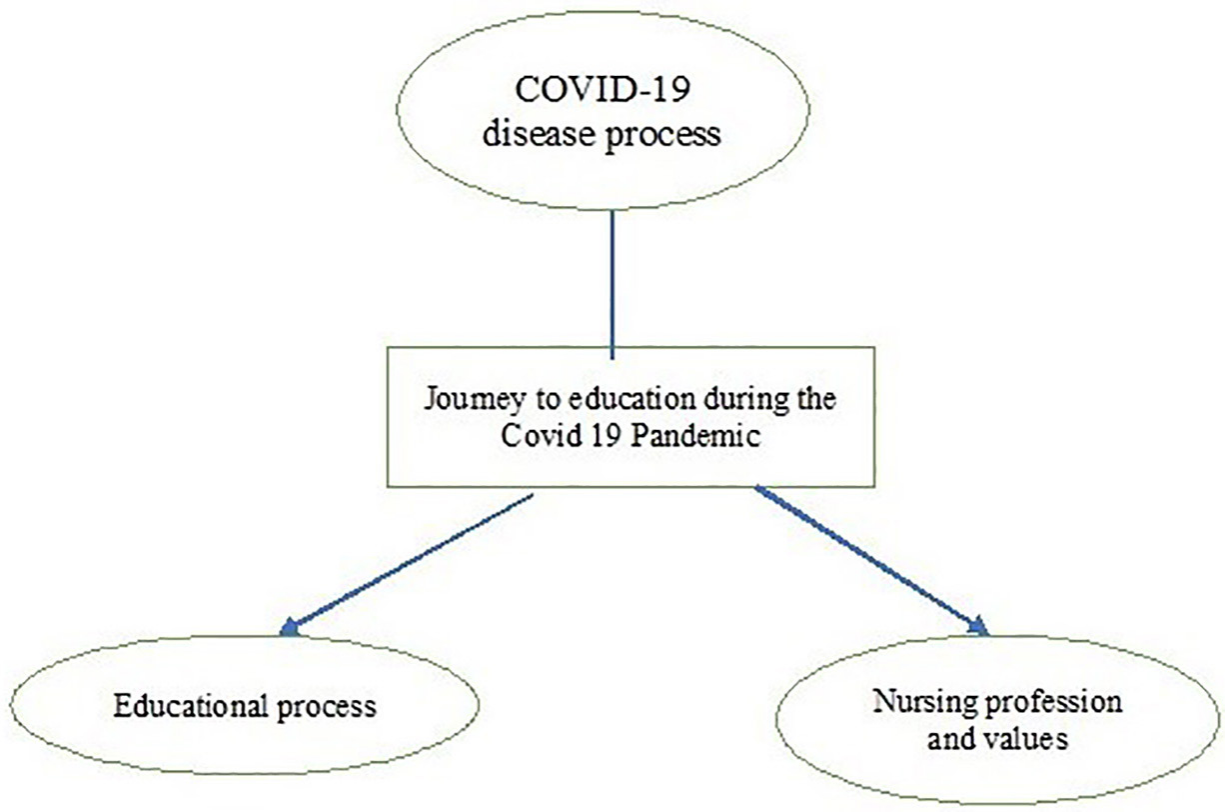
Representation of themes in the experiences os nursing students who contracted Covid-19 during face-to-face education.

In this study, the experiences of those nursing students who contracted Covid-19 during face-to-face education were examined. Eleven nursing students participated in the research; nine of the students were female, with an average age of 22.7. Six of them were fourth-year students, with a general grade point average of 3.20 out of 4. Seven students resided in student dormitories, and nine had family members who had contracted Covid-19. All participants stated that they had received two doses of the Covid-19 vaccine. A total of 269 primary codes were identified through MAXQDA 20 analysis in the study. The descriptive analysis resulted in the categorization of codes under three themes. These are COVID-19 disease process, educational process, and nursing profession and values (Figure 1).

The themes “Covid-19 disease process”, “Educational process”, and “Nursing profession and values” were established, each with associated subthemes (Chart 1).

**Chart 1:** Themes and subthemes – Çanakkale, Turkey, 2022.

COVID-19 disease process	Educational process	Nursing profession and values
Emotions; fear, anxiety	Technological problems, financial constraints	Importance of nursing
Gains; socialization, appreciation of health	Legal issues	Intense and challenging working environment

### Result 1. Theme of Covid-19 Disease Process

Within the theme of the Covid-19 disease process, two subthemes emerged: “emotions” and “gains”.

Categories of the emotion subtheme are; trust in the vaccine, distrust in the vaccine, exaggeration of Covid 19, burnout, fear, stress, disease process, difficulties, using a mask, hygiene, loneliness, treatment process, uncertainty, isolation, symptoms, fear while waiting for the test result, anxiety, being isolated, loneliness, fear of being positive, feeling sad about being positive, overthinking, not being able to recover, anxiety, getting used to the situation, uncertainty, being produced in a laboratory. Categories of the gains subtheme are; socialization, importance of health, gratitude, importance of social support and hygiene, family, friends.

During the initial stages of the Covid-19 pandemic, the students expressed feelings of anxiety and fear due to the uncertainty surrounding the effects of the disease and the implementation of restrictions. They mentioned being more attentive to hygiene and mask usage. However, after the discovery of the Covid-19 vaccine and the gradual easing of restrictions, coupled with the spread of news about the mild symptoms of the disease, the participants reported a decrease in their fears and concerns. They also noted that they were not as vigilant about mask usage as before, leading to illness. The participants predominantly expressed views related to “fear and loneliness” and “social support” within the category of the disease process:

“I never thought I had Covid-19 because I was doing an internship in the vaccination unit. As far as I knew, those who had Covid-19 weren’t coming in for vaccinations. But I got a severe case of flu; I thought it was just a common cold, but I felt really bad and went to the hospital. They tested me, and the result came back positive. I isolated myself, separated my room. For a week, I didn’t do anything; I had a lot of pain, and I spent the time alone in my room with pain and worry. There was complete uncertainty; I didn’t know anything. Fear inevitably envelops you. What will happen, when will I recover, will it be a permanent illness, I had a great fear and uncertainty about having children in the future, to be honest, I still feel uncertain.” (P9)

“When schools reopened during the pandemic, I was very scared; I had panic attacks with the fear of getting Covid-19. When I found out I had Covid-19, I had panic attacks; I was taken to the emergency room a few times. Covid-19 was truly traumatic for me; I don’t want to remember those days.” (P11)

“I spent it alone at home, it was very bad, I was all alone, I couldn’t even go shopping, and no one did shopping for me either. My morale was very low. Throughout the illness, I didn’t feel safe. I even experienced the fear of death; I wish I had the opportunity to see a therapist.” (P7)

The students expressed skepticism about the origin of the Covid-19 virus, stating that they no longer trusted the authorities’ statements. All of the students stated that they had received at least two doses of the vaccine. Some mentioned that the vaccine helped them experience milder symptoms and considered it effective. However, the others expressed doubts about the vaccine’s effectiveness, stating that they did not trust it and believed that it did not provide protection. They mentioned having received both the vaccine and still contracting Covid-19. The students noted that getting the Covid-19 vaccine was mandatory to attend clinical placements, even if they were hesitant or did not want to get vaccinated. Some attributed their lack of trust in the vaccine to the abundance of anti-vaccine articles and videos on social media and the internet, as well as their families’ negative attitudes toward vaccination.

“COVID-19 vaccines were produced and released so quickly that I really couldn’t trust them at all.” (P11)

“I had to get vaccinated to do my internship. I didn’t want to get vaccinated at all. In fact, my family and I are anti-vaxxers. There are many side effects associated with the vaccine written on social media, so I don’t trust that the vaccine is harmless.” (P6)

In the category of “gains”, the students concentrated on the codes of “socialization” and “hygiene”. With COVID-19, they expressed that they learned the value of being able to breathe freely, socialize, and be close to their loved ones, as well as the importance of health. The students emphasized the importance of cleanliness during this period.

“What did Covid-19 teach me? We had to stay away from our loved ones by necessity ... I think it taught us a lot, especially about hygiene. Especially when I used to come home, I didn’t pay attention to washing my hands, but now I wash them all the time, I’m very careful, washing hands has become an obsession for me.” (P2)

“Covid took away our social life, that was the biggest disadvantage. I realized the importance of socializing very well. It was impossible to go out and get some fresh air, there was a constant lockdown, especially young people and the elderly were very affected by the restrictions in my opinion. Not being able to meet my friends, staying at home for days on end was like a nightmare, it really pushed us.” (P7)

“We remembered how wonderful it was to breathe and to have a sense of smell because we constantly wore masks. I dreamed of being able to breathe freely. We also understood the value of that. There is always a lesson to be learned from every bad thing, I hope everyone in the world has learned a lesson, we appreciate our breath. I was very grateful when I recovered. Because I had lost my sense of smell, it was horrible for me, but three months later I started to smell again.” (P11)

It can be considered that receiving psychosocial support during the isolation process can evoke positive feelings in individuals, and they may cope better mentally than those who go through the isolation process alone. It is crucial for individuals not to be left alone during the isolation process and to receive both financial and emotional support. If necessary, online psychological support should be provided in such situations. Additionally, it is important for school management and teachers to make Covid-19 positive students feel that they are not alone by contacting them online or by phone during the isolation process.

### Result 2. Theme of Educational Process

In the theme of the educational process, two sub-themes emerged: “technological problems” and “legal issues”. Categories of technological problems are; not having a personal computer, not being able to access the internet, inefficiency of online education, decrease in the grade point average of some participants and increase in others. Categories of legal issues, systemic problems, not being able to submit homework on time while in isolation, being unprepared for the pandemic, not being able to take exams, falling behind in courses and practices.

The participants expressed that both students and teachers experienced problems related to technology. They mentioned that students’ focus on the lessons decreased because they could not make eye contact with their teachers, and they often listened to the lessons like radio broadcasts. The participants also stated that they could not discuss the lessons face-to-face with their friends and that there was no attendance requirement in online classes, which made the education during the pandemic ineffective. The students reported experiencing numerous problems in online classes and exams due to the system frequently crashing.

“I think distance education had a lot of shortcomings. The teachers didn’t know how to use the system, and they struggled a lot because they weren’t used to remote teaching, especially for practical lessons. Normally, classes were 20 minutes long, but the teachers tried to make the class hours longer. It felt like listening to a radio broadcast, but it still wasn’t enough for practical lessons. Nobody was prepared for this situation; our internet data was running out, and the internet was very expensive. It was really challenging, to be honest.” (P3)

“We faced the following problems online: I didn’t have a personal computer, and all three of my siblings were also receiving online education at home. We were in the village, where the internet connection was poor. I sent several exams late because we were in the village, and the internet was very problematic; sometimes it worked, sometimes it didn’t. My siblings were studying from the television; they were in primary school. Our house had a stove, and my mother was very tired. Online education also affected our family a lot; we tried to live in a tiny room with a stove, and we faced a lot of difficulties.” (P6)

“Not everyone had a computer or internet at home; we used to communicate with our friends through groups. This was a huge disadvantage, and at the same time, it was easy to cheat, but my grades dropped during the pandemic. My GPA was higher in face-to-face education. I never liked online education; hopefully, there won’t be such a thing again.” (P7)

The students who contracted Covid-19 close to exam time expressed experiencing stress due to exam anxiety.

“My isolation ended, and I immediately entered the exams, which caused me extreme stress. I didn’t know what to do in the exams because I couldn’t attend classes.” (P11)

“Online education was not good at all. I couldn’t connect to the internet because we live in the mountains, so whenever it rains or something happens, the electricity goes out immediately. The infrastructure of the village is very poor, so the internet would cut off quickly. During school classes, you learn a lot by discussing with friends and studying together for exams. But during the pandemic, we just memorized the presentations given by the teachers and took the exams; the information didn’t stick, unfortunately. During exam week, I entered an exam, but the internet cut off immediately, and I had to withdraw from another exam at the last minute because the internet went out. So, I went through very stressful, difficult, and bad times. I never want online education again.” (P8)

Some students expressed difficulties due to lack of personal computers and limited internet access in their geographical region. They particularly highlighted stress during exams due to internet-related issues. With the onset of Covid-19, the students found themselves dealing with both the stress of illness and challenges in online education. Disparities in socioeconomic status and living conditions meant that while some students had easy access to educational tools (computers, tablets, phones), the others faced financial constraints and lacked access to necessary technology due to the technological infrastructure of their region. This situation led to educational inequality. Additionally, the students reported being unable to derive benefit from online classes, inability to interact with peers, spending entire days at home, and the uncertainty of the situation, all of which contributed to stress. Despite the circumstances, the students repeatedly emphasized that face-to-face education would be more effective, especially for practical vocational courses that simply cannot be conducted online.

### Result 3. Theme of Nursing Profession and Values

In the theme of nursing profession and values, two sub-themes emerged: “importance of nursing” and “intense and challenging working environment”. The categories of the importance of nursing are; loving the profession positively, being proud, being happy to study nursing, awareness, valuing the profession, Intense and challenging working environment categories, workload, risks, fear, difficulty, not being accepted to the practice, the adaptation process after the disease, fear of getting Covid again, being cautious towards everyone, anxiety.

The participants highlighted that nurses worked under difficult conditions during the Covid-19 pandemic, emphasizing that they are the backbone of the healthcare system. They noted that the need for the nursing profession increased with the pandemic, stressing that both healthy and sick individuals rely on nurses. The students mentioned that, although they initially entered nursing school reluctantly, the Covid-19 pandemic made them realize the significance of nursing and helped them develop a passion for the profession. They expressed happiness in helping others and acknowledged nurses as true heroes.

“ I graduated from high school through online education during the Covid-19 pandemic in 2020, and I also took the university entrance exam in the same year. Before taking the university entrance exam, I had no knowledge about fields like nursing, but I always wanted to work in the healthcare sector. It’s ironic that I graduated from high school online due to the Covid-19 pandemic. When the university entrance exam results came, a light for nursing lit up, and I decided to choose it. But as I said, I never thought about it before, and I didn’t really want to pursue it, to be honest. During the pandemic, I truly saw how heroic and selfless healthcare workers are. I mean, considering the workload, nurses’ salaries are already quite low. Despite that, nursing is a truly sacred profession with a very high spiritual value. They left their families at home and took care of patients during the pandemic. Is there anything beyond that? For me, it’s fulfilling. I’m glad I chose nursing.” (P11)

“I had a lot of prejudices about nursing, and I came to this department unwillingly. I thought it was a very tiring profession because I have relatives who are nurses, and we see how exhausted they are. However, when I entered this profession, my prejudices were shattered. I came here reluctantly, but I ended up loving it. I realized that I love nursing when I was doing my internship at the hospital, but the working conditions are very challenging.” (P10)

The uncertainty that began with the pandemic led to other fears among the students with the transition to face-to-face education. The students who continued their clinical practices alongside face-to-face education expressed hesitation in approaching patients due to the fear of getting re-infected after contracting Covid-19. The reasons for this may include the uncertainty of the Covid-19 disease, inability to attend school, loneliness, social isolation, exam anxiety, lack of internet access, and the possibility of the disease progressing more severely.

## DISCUSSION

In this study, the experiences of nursing students who contracted Covid-19 during face-to-face education amid the Covid-19 pandemic were examined. Based on the data obtained from the nursing students, the themes Covid-19 disease process, educational process, and nursing profession and values were identified.

In the Covid-19 pandemic, universities transitioned to online education. Students in practical fields such as nursing were significantly affected during this period both psychologically and in terms of skill development. In a study conducted with students from the Faculty of Health Sciences, it was found that participants had a moderate level of fear related to Covid-19. Changes in the level of Covid-19 fear were found to be associated with factors such as gender, thoughts on Covid-19 transmission, getting tested, isolation, following developments related to Covid-19, using protective equipment, adhering to social distancing, and prioritizing hygiene^(19)^. In a study examining the risk perceptions, fears, depression, anxiety, stress, and coping mechanisms of Saudi nursing students during the Covid-19 pandemic, it was found that students’ limited knowledge of Covid-19, high perceived infection risk, and certain coping strategies were associated with depression, anxiety, stress, and fear among students^(20)^. Another study conducted with health sciences students found that even a year after the Covid-19 pandemic, students continued to experience stress and anxiety related to Covid-19^(21)^.

In this study, it was expressed that the students who had Covid-19 experienced anxiety primarily due to fear and uncertainty. The prolonged duration of test result disclosure is thought to be attributed to the participants’ high levels of concerns. The participants’ worries about unintentionally transmitting the disease to others and the uncertainty of the illness process led to negative emotional states. During isolation, the lack of social interaction with family and friends caused stress among the participants. It is thought that psychological support is necessary during social isolation. In a study investigating the effect of social support on stress among university students during the Covid-19 pandemic, it was found that social support had a positive effect in reducing stress among students who were isolated due to Covid-19^(22,23)^. Participants reported exhibiting hesitant behaviors when approaching patients during face-to-face clinical practices amid the Covid-19 pandemic due to fear of reinfection. Possible reasons for this behavior may include the uncertainty surrounding Covid-19, inability to attend school, social isolation, exam anxiety, lack of internet access, and the possibility of the disease progressing more severely.

The students emphasized the importance of socialization and the value of health in terms of their gains. They expressed that after contracting Covid-19, they learned the importance of adhering to all rules, especially wearing masks, maintaining distance, and practicing good hygiene, in order to avoid losing their health.

Some students expressed their reluctance to get the Covid-19 vaccine and stated that they did not trust it. The reason behind this reluctance is thought to stem from the abundance of anti-vaccine information and videos on social media and the internet, leading to feelings of distrust among individuals due to the infodemic effect. Literature review supports this research. In a study investigating the attitudes of medical students towards the Covid-19 vaccine, it was found that students had a positive attitude towards the Covid-19 vaccine^(24)^. In a survey conducted in Turkey with 1293 participants regarding attitudes towards the Covid-19 vaccine, 41.2% responded positively to getting vaccinated. Among those who were unwilling to get vaccinated, 75.9% feared the side effects of new vaccines, 34.4% did not trust the companies producing the vaccine, and 20.9% did not believe in the effectiveness of the vaccine. The study concluded that there was “hesitancy” towards the Covid-19 vaccine^(25)^. Another researcher examined 118 YouTube videos about the Covid-19 vaccine. The study found that 42.4% of the videos supported the vaccine, that 19.5% were against it, and that 38.1% were neutral. It was also found that videos against or neutral towards the vaccine had more views compared to videos in favor of the vaccine^(26)^. It can be inferred that there is still distrust towards the Covid-19 vaccine in society today.

In the context of the education process, the students expressed that classes were inefficient, that they faced problems related to internet connectivity, and that some students identified the lack of access to computers as a major issue. However, they also mentioned that during the pandemic, their grades in online exams improved. In a study examining the problems experienced by nursing students during the Covid-19 pandemic, it was found that nursing students, being required to participate in clinical internships alongside theoretical classes, were one of the groups most affected by the pandemic. The study highlighted that students faced intense stress due to the pandemic while also struggling with the educational process. The problems encountered by nursing students during the Covid-19 pandemic were categorized as “issues with the university’s distance education infrastructure”, “lack of face-to-face education”, “personal computer limitations, financial constraints”, “emotional impact of the pandemic”, and “exam anxiety”^(5)^. In another study focusing on nursing students during the pandemic, it was reported that various challenges were faced, such as the inability to attend clinical training, difficulty accessing information during remote learning, inability to attend classes due to the cost of the internet, lack of internet access due to the geographical location, and insufficient technology at home, such as inadequate computers or mobile phones, especially in households with multiple students, leading to disruptions in education and inequalities^(6)^. During the pandemic period, students’ positive thoughts towards nursing education were effective in developing positive attitudes towards distance education; Uncertainty regarding distance education and the decreased perception of learning due to the Covid-19 epidemic cause negative attitudes towards distance education^(27)^.These findings support the results of our study. Many students also mentioned the difficulties of having siblings also receiving online education, inadequate physical conditions at home, and internet issues. Additionally, the students stated that instructors were unprepared for online teaching. In a study where a scale measuring attitudes towards face-to-face education during the Covid-19 pandemic was developed, it was found that the deficiencies in education, inequalities in educational opportunities, problems faced by teachers, and particularly the uncertainty of the pandemic process were effective in the return to face-to-face education^(28)^.

In the context of nursing profession and values, the students expressed that nurses were the backbone of the healthcare system during the Covid-19 pandemic, working under very challenging and intense conditions, and that they aspired to proudly pursue nursing in the future. In a study, nursing students indicated their willingness to assist in combating the Covid-19 pandemic and their readiness to take on responsibilities despite the extraordinary circumstances, as conveyed through social media platforms^(29)^. Another study conducted online with nursing students during the Covid-19 pandemic revealed that students felt pride in becoming nurses in the future, perceived nursing as an honorable profession, and stated that their families were proud of them^(30)^. In a qualitative study aimed at examining the effects of social isolation during the Covid-19 pandemic on nursing students, interviews were conducted with 49 nursing students. Positive views such as increasing respect and dedication to the profession, the development of unity, and innovative ideas were identified in 31 codes, while negative views such as the challenges, exhaustion, risks, decreasing respect in the profession, and not receiving the deserved value of the profession were identified in 78 codes^(31)^. In a study, it was found that nursing students’ depression and anxiety increased due to social isolation during Covid-19, and they turned to eating to cope with this^(32)^. In Spain, a qualitative study was conducted with 9 nursing students who worked to support nurses in hospitals during the Covid-19 pandemic. The study revealed that nursing students experienced a rapid transition from student to nurse while working to support nurses in hospitals. They expressed fear due to the uncertainty of the situation, tried to remain resilient during the pandemic, developed a sense of belonging to a team, shared a sense of responsibility, and gained an understanding of the importance of the nursing profession^(33)^. It is thought that online education in universities will be inevitable in the future. The experiences of nursing students, as shown in the study, are not only important for nursing education within their own communities but also at the international level.

## LIMITATIONS

The limited number of students included in the study may hinder the generalization of the findings. The interview method used to understand the participants’ experiences may be based on subjective perceptions and may lack objective data.

## CONCLUSION

This study was conducted to determine the experiences of nursing students who received face-to-face education during the COVID-19 pandemic and contracted COVID-19. As a result of the research, three themes were identified; “Covid-19 disease process”, “Educational process”, and “Nursing profession and values”.

1. Covid-19 disease process: Within the theme of the Covid-19 disease process, two subthemes emerged: “emotions” and “gains”. Categories of the emotion subtheme are; trust in the vaccine, distrust in the vaccine, exaggeration of Covid-19, burnout, fear, stress, disease process, difficulties, using a mask, hygiene, loneliness, treatment process, uncertainty, isolation, symptoms, fear while waiting for the test result, anxiety, being isolated, loneliness, fear of being positive, feeling sad about being positive, overthinking, not being able to recover, anxiety, getting used to the situation, uncertainty, being produced in a laboratory. Categories of the gains subtheme are; socialization, importance of health, gratitude, importance of social support and hygiene, family, friends. It is suggested that universities provide psychosocial support to all students affected by the pandemic and monitor its outcomes
2. Educational process: Categories of technological problems are; not having a personal computer, not being able to access the internet, inefficiency of online education, decrease in the grade point average of some participants and increase in others. Categories of legal issues, systemic problems, not being able to submit homework on time while in isolation, being unprepared for the pandemic, not being able to take exams, falling behind in courses and practices. It is recommended that schools update their legal regulations for the possibility of hybrid education in the future. It is also recommended that nursing educators enhance their competencies and receive training in online teaching methods for the hybrid education system.Providing technological support to students is also recommended.
3. Nursing profession and values: The categories of the importance of nursing are; loving the profession positively, being proud, being happy to study nursing, awareness, valuing the profession, Intense and challenging working environment categories, workload, risks, fear, difficulty, not being accepted to the practice, the adaptation process after the disease, fear of getting Covid again, being cautious towards everyone, anxiety. It is recommended that the nursing profession be presented as a professional profession in the media, that the working conditions of nurses be improved, and that nursing, which is at the forefront of all disasters, be highlighted

For future studies, it is recommended to investigate the psychological resilience, attitudes towards the profession, and competencies of nurses who graduated after receiving online education during the Covid-19 pandemic.
